# Differential gene expression analysis in French bulldog high grade oligodendroglioma: breed-associated differences in tumor and tumor microenvironment gene expression

**DOI:** 10.1186/s40575-025-00141-2

**Published:** 2025-05-09

**Authors:** Susan A. Arnold, Juan E. Abrahante Llorens, Jonah Cullen, Eva Furrow, Walter C. Low, G. Elizabeth Pluhar

**Affiliations:** 1 Department of Veterinary Clinical Sciences, University of Minnesota College of Veterinary Medicine, Saint Paul, MN USA; 2 Minnesota Supercomputing Institute, University of Minnesota, Minneapolis, MN USA; 3 Department of Veterinary Population Medicine, Saint Paul, MN USA; 4 Masonic Cancer Center, University of Minnesota, Minneapolis, MN USA

**Keywords:** Canine glioma, French bulldog, Immunotherapy, Transcriptomics

## Abstract

**Background:**

Several canine breeds, including boxers, Boston terriers, and French bulldogs, belong to the same phylogenetic clade and have a higher risk for high-grade oligodendroglioma (HGO) than the general canine population. Despite their shared increased risk for HGO, French bulldogs treated with immunotherapy have experienced worse survival outcomes compared to boxers and Boston terriers. We hypothesized that the French bulldog HGO transcriptome differs from those of boxers and Boston terriers, which might account for the disparity in survival. We performed RNA sequencing on formalin-fixed, paraffin-embedded tissue from French bulldogs, boxers, and Boston terriers to identify differentially expressed genes (DEGs) between French bulldogs and the other evaluated breeds.

**Results:**

There were 31 DEGs in HGO samples from French bulldogs compared to boxers and Boston terriers. Gene set enrichment analysis revealed activated enrichment of 15 cell cycle progression, oncogene, and immune pathways, including E2F targets, mTOR signaling, IL2-STAT 5 signaling, and allograft rejection.

**Conclusions:**

These data confirm the presence of breed-specific canine HGO transcriptomes that can be used to advance our understanding of canine glioma, its translational capacity for human glioma, and precision-based therapies for individual canine patients.

**Supplementary Information:**

The online version contains supplementary material available at 10.1186/s40575-025-00141-2.

## Abstract (plain language summary)

High-grade glioma is a deadly brain cancer that affects pet dogs, especially three related breeds: the French bulldog, boxer, and Boston terrier. Treatment options for pet dogs that develop this devastating brain cancer are limited, and none has yet led to widespread prolonged survival or cure. Thus, a variety of new treatment options are currently being investigated. One of the most promising treatment options is immunotherapy. Immunotherapy recruits the patient’s immune system to fight against the tumor. This treatment has shown promise in other cancers. It has also been investigated in pet dogs with high-grade glioma. However, response to immunotherapy was discovered to be very different between dog breeds. While boxers and Boston terriers respond favorably, French bulldogs responded poorly. We hypothesized that the genetic makeup of French bulldog high-grade glioma tumors differs from those of boxers and Boston terriers and might explain their poor response to immunotherapy. To investigate this hypothesis, we compared gene levels in high-grade glioma brain tumors from French bulldogs relative to those from boxers and Boston terriers. We found 31 genes with different expression levels in French bulldogs compared to in boxers and Boston terriers. These genes play key roles in processes that promote tumor growth and impact immune function. These findings support our hypothesis that French bulldog high-grade glioma is genetically distinct from the tumors of other related breeds. The results of this study increase our understanding of canine brain cancer genetics and might also guide development of more effective therapy.

**Supplementary Information:**

The online version contains supplementary material available at 10.1186/s40575-025-00141-2.

## Background

High grade oligodendroglioma (HGO) is the most common primary, intraparenchymal brain tumor in dogs, accounting for nearly 55% of all canine gliomas [1]. Treatment options are limited for pet dogs with HGO, and outcomes are poor [1–3]. Glioblastoma (GB), a type of glioma in humans, shares many similarities with canine HGO, including devastatingly short survival times [3]. Given the potential for dogs with HGO to serve as a preclinical model for human GB, research investigating the genetic features of these tumors is needed to further characterize the model and to develop novel treatment approaches.

Canine HGO tumors are genetically heterogeneous. One major source of genetic variation is breed. Breed contributes to somatic mutation patterns observed in multiple tumor types [4, 5]. Several studies have characterized the genetic profiles of HGO, but have not included breed as a consideration [6–8]. Characterization of breed-associated HGO gene expression patterns is needed to refine the canine model and also has the potential to reveal differences that correlate with treatment response and prognosis.

There are strong breed predispositions to HGO in dogs. Three breeds that most commonly develop HGO, the French bulldog, boxer, and Boston terrier, fall within a single genetic clade [2, 3, 9]. Yet, clinical behavior of HGO differs between these closely related breeds. In pet dogs with spontaneous HGO enrolled in immunotherapy-based clinical trials, we observed that HGO-bearing French bulldogs had a dramatically shorter median survival time (132 days) than other breeds within the same phylogenetic clade (221 days) [10]. This disparity highlights an opportunity to identify breed-associated differences in HGO that drive poor outcomes. The primary goal of this study was to establish breed-associated transcriptomic profiles for French bulldogs compared to boxers and Boston terriers. We hypothesized that HGO samples obtained from French bulldogs exhibit differences in gene expression relative to the two other clade members. Observed differences will serve as a foundation for exploring contributions to the disparate treatment response of HGO between dog breeds.

## Methods

### Samples and patient population

HGO tissue samples were obtained from biopsy specimens submitted to the Comparative Pathology Shared Resource (CPSR) at the University of Minnesota College of Veterinary Medicine over a 10-year period from 2005 to 2015. Samples from a total of 18 dogs diagnosed with HGO were included. The samples used in this report were obtained via surgical resection as part of enrollment in clinical trials for pet dogs with high grade gliomas. All samples were obtained prior to trial treatment. These studies were approved by the University of Minnesota Institutional Animal Care and Use Committee (IACUC; protocols 2001–37742 A, 2002–37886 A, 2111–39571 A, and 2111–39569 A), and written informed client consent was obtained. Biopsy specimens were submitted to CPSR in 10% neutral buffered formalin and were then embedded in paraffin wax. Hematoxylin and eosin (H&E) stain sections were reviewed by a board-certified veterinary pathologist to confirm a diagnosis of HGO in all cases based on criteria described in the WHO Classification of Tumors of the Central Nervous System [11].

Patient sex, age, tumor location, surgical approach, clinical trial treatment, and progression free survival (PFS) and overall survival (OS) were obtained from each patient’s medical record. Summary statistics were performed to determine the medians and ranges for PFS and OS.

### RNA purification and RNA sequencing

Formalin-fixed, paraffin-embedded (FFPE) samples were used in this study. Previous studies have demonstrated that reliable gene expression data can be extracted from canine FFPE brain tumors [12]. Two 10 μm scrolls were cut from each paraffin block. RNA extraction and RNA sequencing (RNASeq) were performed at the University of Minnesota Genomics Center (UMGC). RNA was extracted and purified from scrolls using the PureLinkTM FFPE Total RNA Isolation Kit (Invitrogen, Carlsbad, CA). After elution of RNA, DNAse I digestion was performed to yield DNA-free total RNA. Total eukaryotic RNA isolates were quantified using a fluorimetric RiboGreen assay (Life Technologies, Carlsbad, CA). Total RNA integrity was assessed using capillary electrophoresis on the Agilent BioAnalyzer 2100 (Agilent, Santa Clara, CA), generating an RNA Integrity Number (RIN). A RIN cutoff of 2 was used for samples to be acceptable. This cutoff complied with the requirement for using the Takara picoMammalian version 2 kit. This kit relies on ribodepletion rather than oligo dt primer to derive polyA tails [13]. This kit was used given that FFPE tissues are expected to be highly degraded, and the Takara picoMammalian kit is optimized for highly degraded RNA samples. Samples that passed quality control underwent library preparation using the SMARTer Stranded Total RNA-Seq Kit v2, Pico Input Mammalian (Takara Bio, Mountain View, CA); this kit removes ribosomal RNA. RNA sequencing was performed on the NovaSeq 6000 S1 flow cell, which produces 2 × 50 bp paired-end reads (Illumina, San Diego, CA) at a targeted depth of ≥ 40 million reads per sample.

RNASeq data were processed by the Minnesota Supercomputing Institute using PURR, a pipeline housed with the Collection of Hierarchical UMII/RIS Pipelines (CHURP). CHURP was developed by a group at the Minnesota Supercomputing Institute, and the analysis is provided as part of the RNASeq package run through the UMGC [13]. Briefly, *2 × 50 bp FastQ paired end reads for 29 samples (n = 61.2 Million average reads per sample) were trimmed using Trimmomatic (v 0.33) enabled with the optional*, *“headcrop − 3” option*, *“-q” option; 3 bp sliding-window trimming from 3’ end requiring minimum Q30.Quality control on raw sequence data for each sample was performed with FastQC. Read mapping was performed via Hisat2 (v2.1.0) using the dog genome (canFam6 NCBI RefSeq assembly GCF_000002285.5) as reference. Gene quantification was done via Feature Counts for raw read counts.*

### Differential gene expression, gene ontology and pathway analysis

Differential expression was tested using the DESeq2 package for R [14]. For this negative binomial model, log2 normalized counts from raw count data were used to determine the log2 fold change estimates. The Wald test for pairwise comparisons was used to measure differences in expression between groups. Dispersion estimates were examined to assess the fit of the data to the DESeq2 model. Principal component analysis (PCA) was performed using log2 transformed fragments per kilobase million (FPKM) values for the 29,993 genes with detectable and variable gene expression. Log2 fold change shrinkage using the apeglm method was used to improve estimated fold changes. Log2 fold change was obtained from the log base 2 of the ratio of median expression values between groups. Differentially expressed genes (DEGs) were identified using the Benjamini-Hochberg adjusted *p*-value for multiple test correction. DEGs were considered statistically significant if they had an adjusted *p*-value < 0.05.

DEGs were classified as either coding or non-coding. Since small, non-coding RNAs, such as snoRNAs and microRNAs can impact RNAseq normalization, these reads were removed [12]. For protein-coding DEGs without a gene name listed, the Ensembl database (https://www.ensembl.org, accessed March 2024) was searched for the Ensembl gene ID to identify its genomic location. This location was then inputted into the University of California Santa Cruz (UCSC) Genome Browser for the Dog10K_Boxer_Tasha/canFam6 genome (https://genome.ucsc.edu/cgi-bin/hgGateway) [15]. A gene name was assigned based on the name of the homologous genes present in the UCSC Genome Browser Non-Dog RefSeq Genes track. The complete list of significant DEGs, including both coding and non-coding DEGs, is provided in Additional file 1.

Gene set enrichment analysis (GSEA) was performed using clusterProfiler in R. The Kolmogorov-Smirnov test, a nonparametric goodness-of-fit test, was used to identify significant gene sets with a *p*-value < 0.05. For each significant gene set, set size, enrichment score (ES), normalized enrichment score (NES), false discovery rate (FDR) and nominal *p*-value were determined. To correct for gene set size and multiple hypothesis testing, the adjusted *p*-values were determined using the Benjamini-Hochberg method, as well as by determining the q-value (or false discovery rate, which had a cutoff of < 0.05 for the screening threshold. To examine the linkages of DEGs, KEGG pathways were examined in clusterProfiler in R as a network using the top 200 DEGs.

## Results

### Patient details

The French bulldog group comprised 11 dogs, and the comparison group comprised 7 dogs, including 4 boxers and 3 Boston terriers. Table 1 summarizes the ages, sexes, and tumor locations for all dogs. The mean age of the French bulldogs was 6.0 years (range: 3 to 9.5 years old), and the mean age of the comparison group was 7.4 years old (range 4-9.5 years old).

**Table 1:** Information about subjects included in the DESeq2 analysis

Dog	Age (years)	Sex	Tumor location
French bulldog 1	5	Neutered male	Left frontal lobe
French bulldog 2	4	Spayed female	Left temporal lobe
French bulldog 3	5.5	Neutered male	Right temporal lobe
French bulldog 4	7	Neutered male	Right parietal lobe
French bulldog 5	9	Neutered male	Left frontal lobe
French bulldog 6	3	Spayed female	Right parietal lobe
French bulldog 7	5	Neutered male	Left frontal lobe
French bulldog 8	4	Spayed female	Left temporal lobe
French bulldog 9	9.5	Spayed female	Left frontal lobe
French bulldog 10	0.75	Spayed female	Left temporal lobe
French bulldog 11	6	Neutered male	Left temporal lobe
Boxer 1	9	Neutered male	Right temporal lobe
Boxer 2	8	Neutered male	Right temporal lobe
Boxer 3	5	Neutered male	Left frontal lobe
Boxer 4	9.5	Spayed female	Right temporal lobe
Boston terrier 1	9.5	Neutered male	Right frontal lobe
Boston terrier 2	6.5	Spayed female	Left parietal lobe
Boston terrier 3	8	Neutered male	Right temporal lobe

### Gene expression profiling

All samples from the 18 dogs passed quality control checks during library preparation and analysis. Over 61.8 million reads were generated per sample. Following read trimming and filtering, rates of genome mapping exceeded 76%, with approximately 10.6 million reads on average matching the Dog10K_Boxer_Tasha/canFam6 transcriptome.

Principal component analysis (PCA) showed that 55% of the variance could be attributed to group (Additional file 2). There was one French bulldog (French bulldog 6) that did not cluster with the rest of the samples in the PCA or hierarchical heatmap (Additional file 3). Medical record review did not identify any clear biological reason why this dog or HGO sample (e.g. storage time, processing, tumor histopathology) differed from the others. Therefore, this French bulldog sample was retained in the DESeq2 analysis. Dispersion estimation to check the fit of the data to the DESeq2 model showed that the majority of the data followed the curve and dispersion decreased with increasing mean (Additional file 4).

**Fig. 1:**
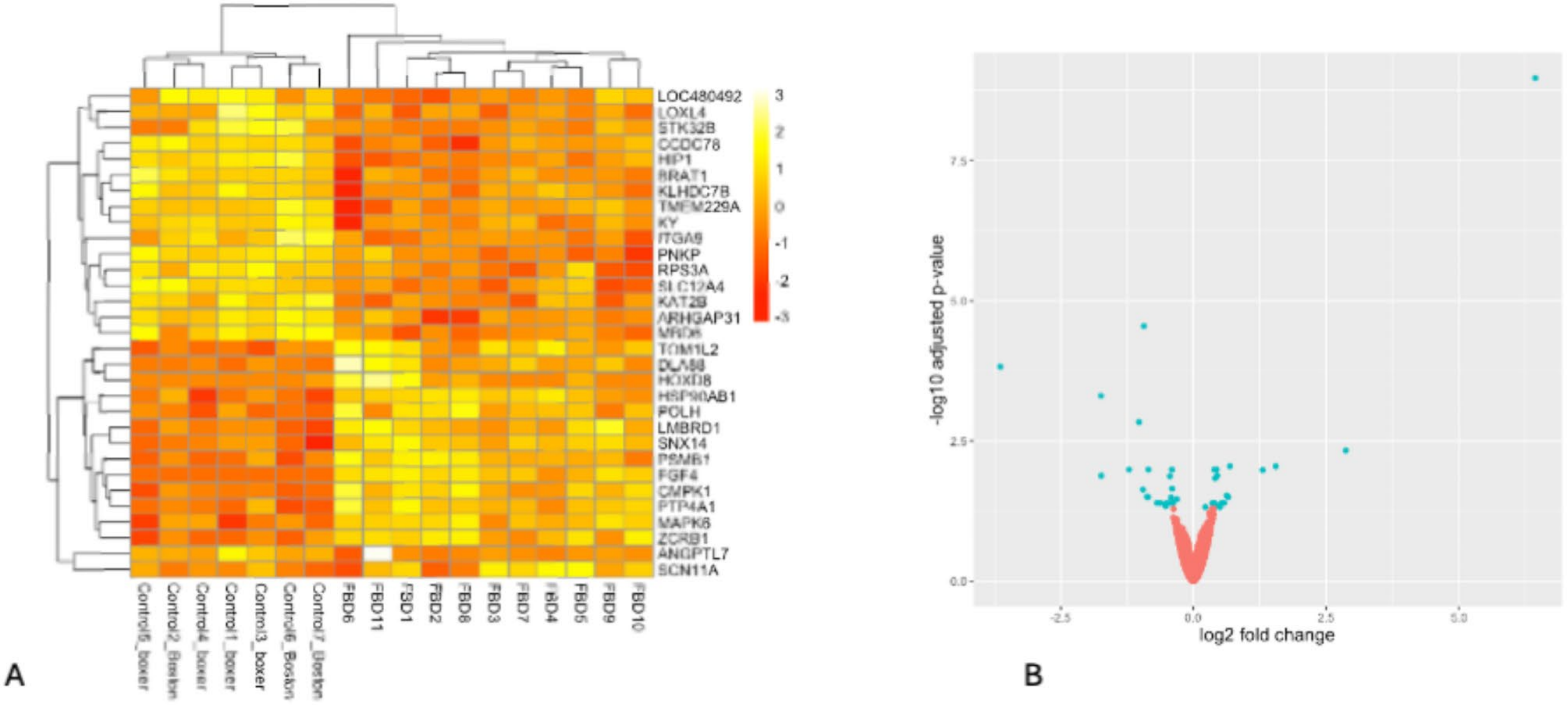
Expression outputs of differential gene expression in French bulldogs versus boxers and Boston terriers. (**A**) heatmap showing the 31 log-transformed genes that were significantly differentially expressed in French bulldogs compared to boxers and Boston terriers. (**B**) volcano plot showing the spread of DEGs. Significant DEGs are identified as blue dots

Differential gene expression analysis with DESeq2 revealed that 45 of the 29,993 annotated genes were differentially expressed in French bulldog HGO tumor tissue compared to the HGO tumor tissue of boxers and Boston terriers (Additional file 1). Of these, 31 were coding DEGs. Fourteen coding DEGs were significantly overexpressed in French bulldogs, and 17 coding DEGs were significantly under expressed in French bulldogs relative to the comparison group (Fig. 1, Tables 2A and 2B).

**Table 2A:** Overexpressed, protein-coding DEGs in French bulldogs compared to boxers and Boston terriers

Gene	baseMean	Log2 Fold Change	lfcSE	*p*-value	*p*-adj
FGF4	14.10	6.44	0.90	5.29E -14	1.07E -09
DLA88	26.40	2.87	1.56	1.39E -06	4.70E -03
HSP90AB1	49.57	0.69	0.42	3.22E -06	9.01E -03
TOM1L2	10.04	0.54	0.59	7.53E -05	4.10E -02
SCN11A	1568.37	0.50	1.20	9.60E -05	4.63E -02
PTP4A1	405.93	0.45	0.18	1.06E -05	1.32E -02
CMPK1	528.43	0.44	0.14	6.41E -06	1.03 E -02
ZCRB1	488.90	0.41	0.13	1.30E -05	1.47E -02
POLH	83.34	0.40	0.24	8.48E -05	4.29E -02
SNX14	740.43	0.40	0.10	4.66E -06	1.03E -02
PSMB1	288.34	0.39	0.15	7.79E -05	4.10E -02
LMBRD1	317.48	0.38	0.14	7.89E -05	4.10E -02
MAPK6	821.43	0.36	0.11	6.38E -05	4.10E -02
HOXD8	38.77	0.23	1.00	1.04E -04	4.81E -02

Summary of the 14 protein-coding DEGs that were overexpressed in French bulldog HGO compared to boxer and Boston terrier HGO. lfcSE = log fold change standard error

**Table 2B:** Underexpressed, protein-coding DEGs in French bulldogs compared to boxers and Boston terriers

Gene	baseMean	Log2 Fold Change	lfcSE	*p*-value	*p*-adj
PNKP	190.80	-0.31	0.08	4.47E -05	3.50E -02
BRAT1	324.00	-0.38	0.12	5.81E -05	4.10E -02
SLC12A4	800.03	-0.40	0.12	2.12E -05	2.26E -02
MBD6	438.46	-0.40	0.11	5.98E -06	1.03E -02
ARHGAP31	2832.03	-0.42	0.20	4.01E -05	3.25E -02
KLHDC7B	52.70	-0.43	0.32	7.82E -05	4.10E -02
KAT2B	3042.61	-0.44	0.16	1.13E -05	1.35E -02
ANGPTL7	19.98	-0.49	0.47	7.09E -05	4.10E -02
RPS3A	133.27	-0.50	0.50	7.14E -05	4.10E -02
TMEM229A	49.29	-0.52	0.65	9.89E -05	4.66E -02
LOC480492	93.60	-0.68	1.02	6.00E -05	4.05E -02
CCDC78	121.07	-0.84	0.58	5.79E -06	1.03E -02
HIP1	3625.95	-0.93	0.30	2.80E-09	2.84E -05
KY	52.16	-1.21	0.85	6.60E -06	1.03E -02
STK32B	16.43	-1.73	2.02	1.03E -05	1.32E -02
ITGA9	1691.35	-1.74	0.48	9.83E -08	4.98E -04
LOXL4	162.56	-3.64	0.85	2.23E -08	1.51E -04

Summary of the 17 protein-coding DEGs that were underexpressed in French bulldog HGO compared to boxer and Boston terrier HGO. lfcSE = log fold change standard error

### Gene set enrichment analysis

**Fig. 2:**
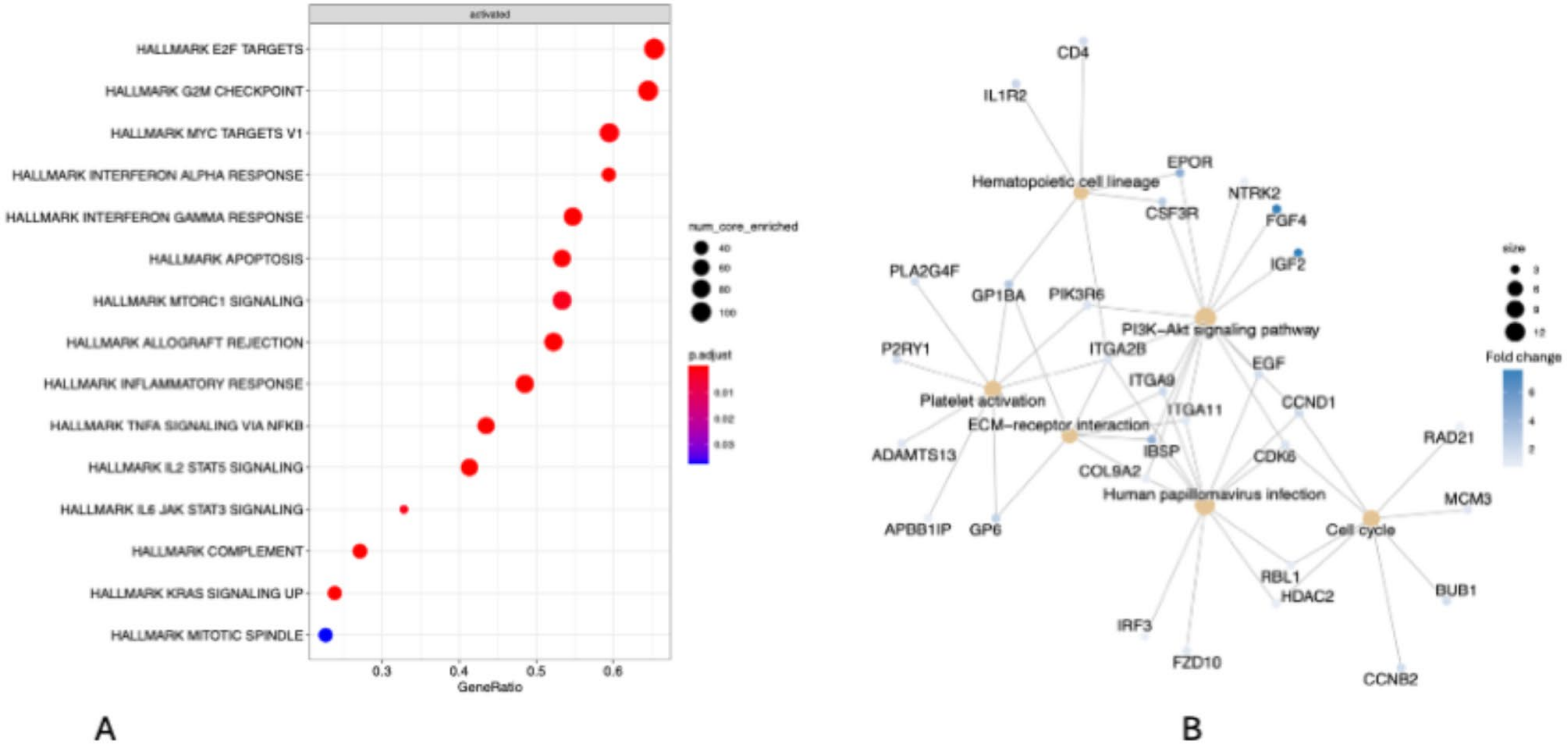
Enriched pathways in French bulldogs compared to boxers and Boston terriers. (**2A**) Dot plot of gene set enrichment analysis, created using clusterProfiler in R. (**2B**) Gene-concept plot depicting linkages of genes and biological concepts using KEGG pathways of the top 200 DEGs, created using clusterProfiler in R

Gene set enrichment analysis (GSEA) revealed 15 significantly enriched gene pathways (Fig. 2A; Table 3). These pathways can be categorized into proteins involved in cell cycle progression (E2F targets, G2-M checkpoint, mitotic spindle), oncogenes (KRAS signaling, mTOR signaling pathway, Myc targets) and proteins involved in immune regulation (IL2-STAT5 signaling, TNFa signaling via NF-kB, interferon gamma response, interferon alpha response, allograft rejection, inflammatory response, complement). The gene concept network plot revealed clusters of genes associated with the PI3K-Akt signaling pathway, the cell cycle pathway, ECM-receptor interaction, and platelet activation, for example (Fig. 2B).

**Table 3:** Enriched pathways in French bulldogs compared to boxers and Boston terriers

Pathway	setSize	ES	NES	pvalue	*p*.adjust	qvalue	rank
Allograft rejection	157	0.74	2.44	1.00E-10	1.25E-09	8.68E-10	1479
Interferon gamma response	148	0.70	2.30	1.00E-10	1.25E-09	8.68E-10	2283
Inflammatory response	167	0.64	2.11	1.00E-10	1.25E-09	8.68E-10	2304
E2F targets	176	0.62	2.05	1.00E-10	1.25E-09	8.68E-10	3638
TNF alpha signaling via NFkB	161	0.61	2.02	8.21E-10	8.21E-09	5.71E-09	2396
G2M checkpoint	172	0.57	1.88	2.15E-07	1.79E-06	1.24E-06	4002
Interferon alpha response	69	0.67	2.00	1.65E-06	1.18E-05	8.19E-06	2796
IL2-STAT5 signaling	167	0.55	1.83	2.34E-06	1.46E-05	1.02E-05	2606
KRAS signaling	172	0.54	1.80	3.07E-06	1.70E-05	1.18E-05	1159
Complement	166	0.54	1.80	3.74E-06	1.87E-05	1.30E-05	1456
Apoptosis	135	0.54	1.74	6.88E-05	3.13E-04	2.17E-04	3626
Myc targets	158	0.50	1.66	1.21E-04	5.05E-04	3.51E-04	4466
IL-6/JAK/STAT3 signaling	64	0.60	1.80	4.31E-04	1.66E-03	1.15E-03	1051
MTORC1 signaling	165	0.49	1.61	5.75E-04	2.05E-03	1.43E-03	3819
Mitotic spindle	181	0.43	1.42	1.13E-02	3.75E-02	2.61E-02	2138

Summary of the 15 enriched pathways in French bulldog HGO compared to boxer and Boston terrier HGO. ES = enrichment score; NES = normalized enrichment score

## Discussion

In this study, we describe the differential gene expression pattern of French bulldog HGO tumor tissue compared to HGO tumor tissue of two related breeds, boxers and Boston terriers, with different tumor behavior. We identified 45 DEGs in HGO transcriptomes that might inform the biological drivers involved in response to immunotherapy. Pet dogs with HGO are an excellent model for human GB, with important similarities life histories, tumor gene expression profiles, tumor-immune interactions, paucity of effective therapies, and amenability to multimodal novel therapeutic investigations [3, 6–8]. Optimization of the model includes establishing breed-associated tumor transcriptomic differences, which may reveal differences in model robustness by breed. This study adds to the emerging characterization of the canine HGO model and our knowledge of how breed impacts differential gene expression and tumor behavior.

Of 14 protein-coding DEGs that were significantly overexpressed in French bulldogs compared to boxers and Boston terriers, some have demonstrated roles in glioma, while others have yet to be evaluated. The most overexpressed DEG in French bulldogs was fibroblast growth factor 4 (FGF4), with a log2 fold change of 6.43 (fold change 86.22). Overexpression of the receptor, FGFR4, has been observed in human glioma tissue compared to normal brain tissue, and overexpression is associated with poor prognosis and faster tumor recurrence. French bulldogs carry one of the two canine FGF4 retrogenes with an extremely high allele frequency of > 90% [16]. Contrastingly, neither retrogene is observed in Boston terriers and has not been evaluated in boxers [16]. Several other dog breeds that do not have a breed-associated risk for HGO have high allele frequency for the same FGF4 retrogene, including beagles, dachshunds, and most spaniel breeds [16, 17]. Thus, overexpression of FGF4 observed in our French bulldog population might merely be a breed-associated variant rather than a feature of their HGO, or it might be possible that this difference in genetic background contributes to more aggressive tumor behavior in dogs that develop HGO.

Other notable overexpressed DEGs included dog leukocyte antigen 88 (DLA88), HSP90AB1, and PTP4A1. DLA88, a major histocompatibility complex (MHC) class I molecule, presents antigens to cytotoxic CD8 positive T cells [18, 19]. Glioma cells can upregulate MHC class I molecules to evade natural killer (NK) cell detection [20, 21]. Given the inflammatory tumor microenvironment, an increased number of infiltrating immune cells, including macrophages, as well as fibroblasts and endothelial cells could also contribute to the observed overexpression of DLA88 in French bulldogs [22]. Human leukocyte antigen (HLA) molecules, analogous to canine DLAs, display a high degree of polymorphism. An individual cancer patient’s HLA profile not only impacts the person’s cancer risk, but also the tumor responsiveness to therapy [23, 24]. Some HLA polymorphisms are associated with favorable responses to immunotherapy, while others are associated with unfavorable responses to immunotherapy [23]. For example, HLA-A*03, observed in 2–16% of the United States population, is associated with reduced overall survival in patients treated with immune checkpoint inhibition [25]. Like human MHC molecules, DLA88 is a highly polymorphic gene, with 139 alleles having been identified and reported to date across breeds [18, 26, 27]. Although the DLA88 profile has not been specifically characterized in French bulldogs or Boston terriers, it has been characterized in boxers [28]. Boxers have been shown to have a single, dominant allele (DLA-88*03401), hypothesized to be the result of severe genetic bottlenecking in the formation of the boxer breed [28]. Further characterization of the DLA88 allelic profile of French bulldogs and Boston terriers and comparison between the three breeds studied herein could further reveal key differences that refine breed-specific canine models of glioma. Additionally, allelic differences in DLA88 in French bulldogs may contribute to the poor response to immunotherapy observed in the present patient population.

HSP90AB1 belongs to the family of heat shock proteins (HSPs), which are involved in antigen presentation, hormonal receptor assembly, protein folding, and cell trafficking in response to cellular stress [29]. An integrated omics analysis predicted HSP90AB1 as a key HSP in glioma [30]. It has also been observed to have a negative association with prognosis and promotes tumor proliferation, migration, and glycolysis [31]. PTP4A1 belongs to a family of liver cell regeneration phosphatase factor proteins and plays an important role in tumor development [32]. PTP4A1 is overexpressed in human glioma, and inhibition of PTP4A1 through microRNA (miRNA) 339-5p abrogates angiogenic mimicry, migration and invasion in a glioma cell line [33]. Elucidating the cellular drivers of overexpression of these DEGs in French bulldogs is an important next step.

There were several DEGs that were overexpressed in the French bulldogs that are not specifically implicated in glioma but belong to important gene families with known roles in glioma, including SCN11A, MAPK6, HOXD8, and PSMB1. Voltage-gated sodium channels, such as SCN11A, are gaining interest as therapeutic targets in human HGG [34–36]. MAPK6 is a member of the mitogen-activated protein kinase (MAPK) signaling pathway. Nearly 50% of MAPK signaling pathway genes are overexpressed in human glioma [37]. While MAPK6 has not been directly implicated in glioma, it promotes tumor growth in several other tumor types, including lung adenocarcinoma, mesothelioma, uveal melanoma, and breast cancer [38]. HOXD8 belongs to the family of genes that encode homeodomain-containing transcription factors that function during embryogenesis to establish cell and tissue identity [39]. In people, HOX A9, A10, C4, and D9 display significant deregulation in GBM tissue. PSMB1 is a member of the proteasome beta family of genes, which encode the beta subunits of the 20 S proteasome. In human glioma tissue, expression of another member of this family, PSMB2, is higher compared to normal brain and correlates with poor prognosis and high tumor grade [40]. Since MAPK6, HOXD8, and PSMB1 all belong to gene families with important roles in glioma, they remain considered targets for future evaluation as canine-specific drivers of HGO.

Of the 17 DEGs that were under expressed in French bulldogs compared to boxers and Boston terriers, some might be protective genes that contribute to the better prognosis in these breeds. For example, PNKP is a DNA repair gene, and BRAT1 interacts with the tumor suppressor gene BRCA1 [41, 42]. Thus, these genes might directly limit tumor growth by reducing mutagenesis. Other under expressed DEGs might play an indirect role in tumor behavior by enhancing response to immunotherapy or might simply represent breed-specific patterns unrelated to tumor behavior.

Of the 15 pathways that were significantly enriched in French bulldogs compared to boxers and Boston terriers, several are important cell cycle progression pathways with known roles in glioma. An often altered pathway in glioma is the p53 tumor suppressor pathway, which is disabled in nearly 80% of human GBM cases [43]. Two prominent downstream targets, E2F targets and the G2M cell cycle checkpoint, are strong hallmarks of tumor proliferation in human glioma, and were significantly enriched in French bulldogs compared to boxers and Boston terriers [43]. G2M arrest is a therapeutic target to inhibit glioma cell growth [44, 45]. These observations validate enrichment of these cell cycle progression pathways as potential drivers of their French bulldog-specific HGO.

In addition to cell cycle regulation pathways, several oncogene pathways were also enriched in French bulldogs compared to boxers and Boston terriers, including KRAS signaling, the mTOR signaling pathway, Myc targets, and IL2-STAT5 signaling. Collectively, these pathways promote cell growth, division, and differentiation [46, 47]. When altered, they act as important oncogenes for nearly all tumor types. Interestingly, previous studies have not demonstrated elevated expression of mTOR in canine glioma cell lines [8]. By comparison, the enrichment of mTOR signaling in French bulldogs disclosed in this study highlights another possible driver of biological differences in the HGO of French bulldogs relative to other breeds. Myc has been observed to be amplified in 78% of canine gliomas [7]. In human glioma, Myc expression correlates with glioma grade [48]. The impact of STAT5 in canine HGO has not been previously identified, but has been named as a potential therapeutic target in canine mastocytoma [49].

The final group of enriched pathways can be categorized as those involved in immune regulation, including TNF-α signaling via NF-kB, interferon gamma response, interferon alpha response, allograft rejection, inflammatory response, and complement. The inflammatory hallmarks TNF-α signaling via NF-kB, allograft rejection, and complement have been observed to be enriched in human glioma tissue [50, 51]. NF-kB is a prominent transcription factor in glioma transformation, growth, angiogenesis, invasion, survival, and therapeutic resistance [52]. Alteration of the key DNA methylation protein, DNMT3A, leads to increased proliferation and malignancy of human glioma through the TNF-α-NF-kB signaling pathway [53]. Glioma cells secrete IL-8 and CCL2 and induce glioma-associated macrophages (GAMs) to secrete TNF-α, which induces endothelial cell activation to promote tumor survival. TNF-α inhibition prolonged survival in a mouse glioma model [54]. The allograft rejection (AR) pathway has been named as a crucial signaling pathway in both low and high grade human glioma and correlates negatively with prognosis [55].

Enrichment of several prognosticating immune-related pathways in French bulldog HGO is an important finding considering the critical relationship between glioma and the immune system and our immunotherapy-treated patient population. One of the most aggressive and treatment-resistant hallmarks of glioma is the manipulation of both the local and systemic immune system. Within the tumor microenvironment, glioma exhibits an “immunologically cold” phenotype, utilizing mechanisms to exclude T cells from the tumor microenvironment and to tolerize and exhaust any infiltrating T cells [56–59]. Glioma also induces an increase in ratios of immunosuppressive phenotypes of macrophages, microglia, myeloid-derived suppressor cells (MDSCs) and regulatory T cells (Tregs) and alters the expression of receptors, costimulatory molecules and cytokines [60]. Overall, the complex array of tumor-immune interactions creates one of the most challenging barriers to treatment. That a large proportion of the enriched pathways observed in French bulldogs were immune-related suggests that French bulldog HGO results in a breed-specific tumor-immune interaction that may impact outcomes in dogs receiving immunotherapy.

A difficulty in comparing the results generated here to previous studies is the rarity of French bulldogs in previous studies of canine HGO transcriptomes [6–8, 61]. It is likely that at the time of these previous studies, the popularity of the French bulldog had not yet soared. Previously a moderately popular dog breed, its popularity exponentially rose recently over a short period of time, becoming the most popular United States dog breed in 2023 [62]. For each of these DEGs, it is possible that over- or under expression is merely a breed-associated variant that is unrelated to HGO development. Comparing the results of this study to the transcriptomes of normal brain tissue in these breeds would help clarify the biological relevance of the observed DEGs.

In our study, the mean age of French bulldogs was lower than the mean age of boxers and Boston terriers (6.0 years versus 7.4 years, respectively). It is possible that some DEGs observed in French bulldogs are associated with tumor formation at a younger age. Further analysis is needed to determine the relationship between tumor DEGs and patient age. Tumor location differed between French bulldogs and boxers and Boston terriers. Most notably, there were more boxers with tumors in the temporal/piriform lobe than French bulldogs. Spatial transcriptomics would be a valuable method to determine if DEGs are restricted to different cerebral lobes and to evaluate regions or normal versus tumor-infiltrated regions. Sex also differed between groups with a greater proportion of females in the French bulldog group. It was not possible to perform matching for age, tumor location, or sex between groups due to a limited HGO sample biobank for these breeds. Finally, although most of the dogs clustered by breed in the principal component analysis, French bulldog 6 did not cluster with the rest of the samples. No clinical or sample handling differences were detected in this dog compared to the others. However, given that breed was designated based on the dog owners’ declarations, it is always possible that the heritage of a dog is different than disclosed. This is one possible reason that French bulldog 6 appeared as an outlier, although this is entirely suppositional. Upon realizing that French bulldogs were doing poorly when treated with immunotherapy, we eliminated them from future trial enrollment due to this ethical consideration, and this limited the samples available from this breed.

## Conclusions

In summary, this study discloses differential gene expression and pathway enrichment in French bulldog HGO compared to boxers and Boston terriers. These data provide a platform for future studies to further characterize breed-specific HGO transcriptomes and to determine the association between observed DEGs and patient outcome.

## Electronic supplementary material

Below is the link to the electronic supplementary material.

Additional file 1: Complete list of all significant DEGs identified in French bulldogs compared to boxers and Boston terriers (This file contains the composite output of the DESeq2 analysis performed to investigate DEGs of French bulldog HGO samples versus boxer/Boston terrier HGO samples. All DEGs, including both protein-coding and non-protein-coding DEGs, are included. For non-protein-coding DEGs, the gene type is further detailed)

Additional file 2: 3-dimensional principal component analysis (PCA) of included RNASeq samples (This is a PCA plot depicting the variance in the data. French bulldog HGO samples are represented as pink dots. Boxers and Boston terrier HGO samples are represented as blue dots. Breed (French bulldog versus boxers/Boston terriers) accounted for 54% of the variance in the data)

Additional file 3: Heatmap of hierarchically clustered sample-to-sample distances (Hierarchical heatmap depicting the clustering of samples by breed. Most French bulldogs cluster together, and most boxers and Boston terriers cluster together, apart from French bulldog 6 (FBD6))

Additional file 4: Dispersion plot of DESEq2 analysis (This is a dispersion plot of the mean of normalized counts across the data set. For the data, as the mean of the normalized counts increases, the dispersion decreases. The majority of genes fit the curve and decrease with increasing mean)

## Data Availability

The datasets generated and analyzed during the current study are available in the Data Repository for the University of Minnesota (DRUM), https://hdl.handle.net/11299/267779. The datasets generated and analyzed are also available in the NCBI Gene Expression Omnibus (GEO) under accession number GSE284559, https://www.ncbi.nlm.nih.gov/geo/query/acc.cgi?acc=GSE284559.

## Abbreviations

DEG: Differentially expressed gene
GSEA: Gene set enrichment analysis
GB: Glioblastoma
HGG: High-grade glioma
HGO: High-grade oligodendroglioma
HSP: Heat shock protein
MAPK: Map kinase
MDSC: Myeloid derived suppressor cell
MHC: Major histocompatibility complex
OS: Overall survival
PCA: Principal component analysis
PFS: Progression free survival
RNASeq: RNA Sequencing
Treg: Regulatory T cell
