# Supplementary material for: Differential gene expression analysis in French bulldog high grade oligodendroglioma: breed-associated differences in tumor and tumor microenvironment gene expression
Source: Companion Anim Health Genet. 2025 May 9;12:4. doi: 10.1186/s40575-025-00141-2 (PMC12063443; doi:10.1186/s40575-025-00141-2)
Supplement: Supplementary file 1 — Additional file 1: Complete list of all significant DEGs identified in French bulldogs compared to boxers and Boston terriers (This file contains the composite output of the DESeq2 analysis performed to investigate DEGs of French bulldog HGO samples versus boxer/Boston terrier HGO samples. All DEGs, including both protein-coding and non-protein-coding DEGs, are included. For non-protein-coding DEGs, the gene type is further detailed) [file 40575_2025_141_MOESM1_ESM.docx]

Additional file 1

Description: Complete list of all significant DEGs identified in French bulldogs compared to boxers and Boston terriers.

| **Gene** | **baseMean** | **log2FoldChange** | **lfcSE** | **pvalue** | **padj** | **Gene type** |
| --- | --- | --- | --- | --- | --- | --- |
| FGF4 | 14.1 | 6.44 | 0.90 | 5.29E-14 | 1.07E-09 | protein coding |
| DLA88 | 26.40 | 2.67 | 1.56 | 1.39E-06 | 4.70E-03 | protein coding |
| ENSCAFG00000050582 | 9.90 | 1.55 | 0.94 | 3.56E-06 | 9.00E-03 | miRNA |
| ENSCAFG00000057673 | 8.00 | 1.31 | 0.96 | 7.24E-06 | 1.00E-02 | processed pseudogene |
| EMC2 | 49.60 | 0.69 | 0.42 | 3.22E-06 | 9.00E-03 | protein coding |
| ENSCAFG00000049640 | 171.80 | 0.66 | 0.65 | 3.68E-05 | 3.20E-02 | lncRNA |
| ENSCAFG00000021808 | 413.60 | 0.63 | 0.57 | 3.12E-05 | 3.00E-02 | snoRNA |
| ENSCAFG00000026472 | 167.94 | 0.58 | 0.61 | 5.62E-05 | 4.05E-02 | snoRNA |
| SNORD34 | 462.48 | 0.54 | 0.53 | 5.57E-05 | 4.05E-02 | snRNA |
| TOM1L2 | 10.04 | 0.54 | 0.59 | 7.53E-05 | 4.10E-02 | protein coding |
| SCN11A | 1568.37 | 0.50 | 1.20 | 9.60E-05 | 4.63E-02 | protein coding |
| ENSCAFG00000025773 | 119.99 | 0.50 | 0.62 | 1.07E-04 | 4.82E-02 | snoRNA |
| ENSCAFG00000025859 | 330.82 | 0.48 | 0.50 | 9.45E-05 | 4.63E-02 | snoRNA |
| ENSCAFG00000002490 | 405.93 | 0.45 | 0.18 | 1.05E-05 | 1.32E-02 | snoRNA |
| CMPK1 | 528.43 | 0.44 | 0.14 | 6.41E-06 | 1.03E-02 | protein coding |
| ZCRB1 | 488.90 | 0.41 | 0.13 | 1.30E-05 | 1.47E-02 | protein coding |
| ENSCAFG00000027581 | 630.42 | 0.40 | 0.22 | 7.12E-05 | 4.10E-02 | snoRNA |
| POLH | 83.34 | 0.40 | 0.24 | 8.48E-05 | 4.29E-02 | protein coding |
| SNX14 | 740.43 | 0.40 | 0.10 | 4.66E-06 | 1.03E-02 | protein coding |
| PSMB1 | 288.34 | 0.39 | 0.15 | 7.79E-05 | 4.10E-02 | protein coding |
| LMBRD1 | 317.48 | 0.38 | 0.14 | 7.89E-05 | 4.10E-02 | protein coding |
| MAPK6 | 821.43 | 0.36 | 0.11 | 6.39E-05 | 4.10E-02 | protein coding |
| HOXD8 | 38.77 | 0.23 | 1.00 | 1.04E-04 | 4.81E-02 | protein coding |
| PNKP | 190.79 | -0.31 | 0.08 | 4.47E-05 | 3.49E-02 | protein coding |
| BRAT1 | 324.04 | -0.38 | 0.12 | 5.81E-05 | 4.05E-02 | protein coding |
| SLC12A4 | 800.03 | -0.40 | 0.12 | 2.12E-05 | 2.26E-02 | protein coding |
| MBD6 | 438.46 | -0.40 | 0.11 | 5.98E-06 | 1.03E-02 | protein coding |
| ARHGAP31 | 2832.03 | -0.42 | 0.20 | 4.01E-05 | 3.25E-02 | protein codin |
| KLHDC7B | 52.70 | -0.43 | 0.32 | 7.82E-05 | 4.10E-02 | protein coding |
| KAT2B | 3042.61 | -0.44 | 0.16 | 1.13E-05 | 1.35E-02 | protein coding |
| ANGPTL7 | 19.98 | -0.49 | 0.47 | 7.09E-05 | 4.10E-02 | protein coding |
| RPS3A | 133.27 | -0.50 | 0.50 | 7.14E-05 | 4.10E-02 | protein coding |
| TMEM229A | 49.29 | -0.52 | 0.65 | 9.89E-05 | 4.66E-02 | protein coding |
| ENSCAFG00000051210 | 466.31 | -0.61 | 0.93 | 7.44E-05 | 4.10E-02 | lncRNA |
| LOC480492 | 93.60 | -0.68 | 1.02 | 6.00E-05 | 4.05E-02 | protein coding |
| CCDC78 | 121.07 | -0.84 | 0.58 | 5.79E-06 | 1.03E-02 | protein coding |
| ENSCAFG00000058064 | 2.74 | -0.85 | 1.18 | 3.79E-05 | 3.20E-02 | lncRNA |
| ENSCAFG00000053084 | 32.06 | -0.87 | 1.10 | 3.44E-05 | 3.17E-02 | lncRNA |
| HIP1 | 3625.95 | -0.93 | 0.30 | 2.80E-09 | 2.84E-05 | protein coding |
| ENSCAFG00000055638 | 8.91 | -0.95 | 0.97 | 2.33E-05 | 2.36E-02 | lncRNA |
| ENSCAFG00000053379 | 30.30 | -1.02 | 0.47 | 3.62E-07 | 1.47E-03 | lncRNA |
| KY | 52.16 | -1.21 | 0.85 | 6.60E-06 | 1.03E-02 | protein coding |
| STK32B | 16.43 | -1.73 | 2.02 | 1.03E-05 | 1.32E-02 | protein coding |
| ITGA9 | 1691.35 | -1.74 | 0.48 | 9.83E-08 | 4.98E-04 | protein coding |
| LOXL4 | 162.56 | -3.64 | 0.85 | 2.23E-08 | 1.51E-04 | protein coding |
