# Supplementary figures and images for: Differential gene expression analysis in French bulldog high grade oligodendroglioma: breed-associated differences in tumor and tumor microenvironment gene expression
Source: Companion Anim Health Genet. 2025 May 9;12:4. doi: 10.1186/s40575-025-00141-2 (PMC12063443; doi:10.1186/s40575-025-00141-2)

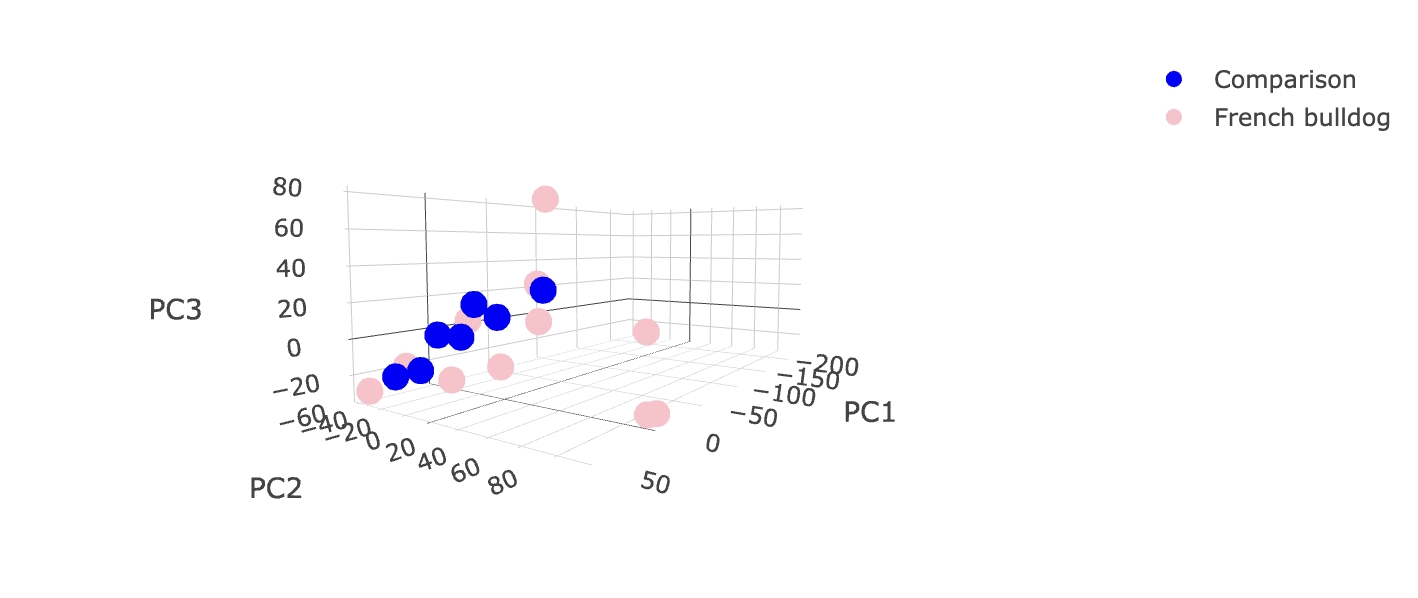

Supplement: Supplementary file 2 — Additional file 2: 3-dimensional principal component analysis (PCA) of included RNASeq samples (This is a PCA plot depicting the variance in the data. French bulldog HGO samples are represented as pink dots. Boxers and Boston terrier HGO samples are represented as blue dots. Breed (French bulldog versus boxers/Boston terriers) accounted for 54% of the variance in the data) [file 40575_2025_141_MOESM2_ESM.jpeg]

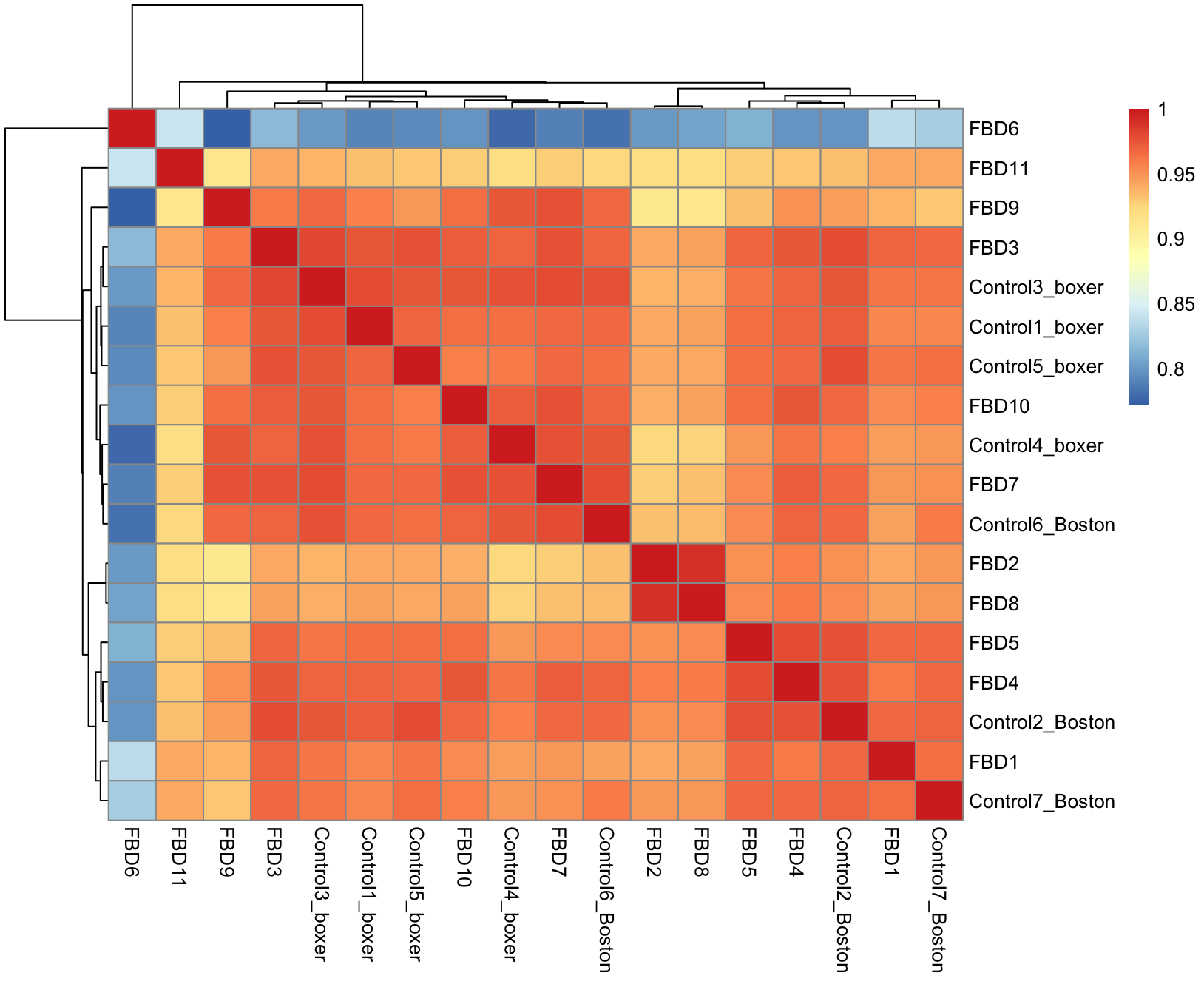

Supplement: Supplementary file 3 — Additional file 3: Heatmap of hierarchically clustered sample-to-sample distances (Hierarchical heatmap depicting the clustering of samples by breed. Most French bulldogs cluster together, and most boxers and Boston terriers cluster together, apart from French bulldog 6 (FBD6)) [file 40575_2025_141_MOESM3_ESM.png]

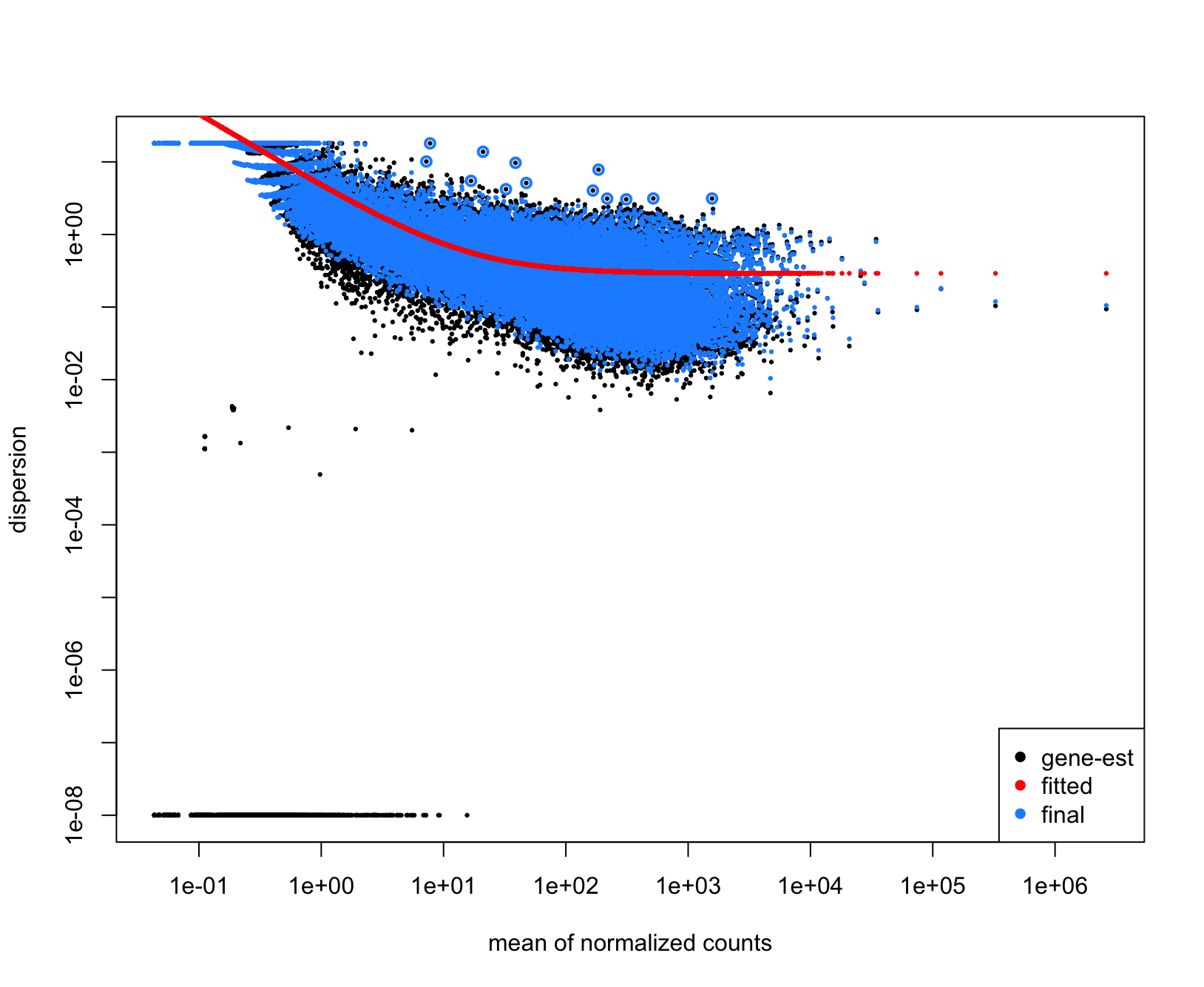

Supplement: Supplementary file 4 — Additional file 4: Dispersion plot of DESEq2 analysis (This is a dispersion plot of the mean of normalized counts across the data set. For the data, as the mean of the normalized counts increases, the dispersion decreases. The majority of genes fit the curve and decrease with increasing mean) [file 40575_2025_141_MOESM4_ESM.png]
